# First case of *Phthirus pubis* and *Demodex* co-infestation of the eyelids: a case report

**DOI:** 10.1186/s12886-021-01875-w

**Published:** 2021-03-06

**Authors:** Yanan Huo, Yanping Mo, Xiuming Jin, Xiaodan Huang, Wei Chen

**Affiliations:** 1 Department of Ophthalmology, The Second Affiliated Hospital of Zhejiang University School of Medicine, Hangzhou, 310009 China; 2 Department of Ophthalmology, Huzhou Third Municipal Hospital, Huzhou, 313000 China; 3 Department of Medical Oncology, Tongde hospital of Zhejiang Province, NO, 234, Gucui Road, Hangzhou, 310012 Zhejiang China

**Keywords:** *Phthirus pubis*, Demodex, Co-infestation

## Abstract

**Background:**

*Phthirus pubis* is an obligate parasite of human beings. Demodex spp. is a much more common parasite of human beings. However, *P. pubis* infestation accompanied by Demodex mite infestation in eye has not been reported.

**Case presentation:**

We report the first case of *Phthirus pubis* and *Demodex* co-infestation on a 48-years-old woman. She presented to the hospital with itching and burning at her right eye for 2 weeks. Slit lamp examination revealed multiple nits and adults of *P. pubis* anchored to both upper and lower eyelashes. Eyelashes were trimmed, moxifloxacin eye ointment and fluorometholone eye drops were initiated daily. However, itching didn’t improve after 2 weeks of treatment. Light microscopy examination of eyelashes revealed infestation with *Demodex*. The patient was treated with lid scrubs with 25% tea tree oil daily for 4 weeks and was completely cured.

**Conclusion:**

Our report shows the importance of an early and comprehensive diagnosis, because both phthiriasis palpebrarum and demodicosis can be confused with blepharitis.

## Background

*Phthirus pubis* is an obligate parasite of human beings. It is mainly detectable in the hair of pubic, rectal and inguinal areas. The infestation of *P. pubis* in eyelid is rare, unilateral infestation is extremely rare [1]. Transmission of *P. pubis* to eyelashes may be manual from the infested body hair or during sexual contact. Indirect transmission through clothes or towels contaminated with nits is less frequent.

*Demodex spp.*, on the other hand, is a much more common parasite of human beings. Out of many *Demodex* species, only *Demodex folliculorum* and *Demodex brevis* can parasitize in human eye. *D. folliculorum* is most commonly found in eyelash follicles, whereas *D. brevis* colonizes in the sebaceous and meibomian glands [2]. However, *P. pubis* infestation accompanied by *Demodex* mite infestation in eye has not been reported. Here, we describe the first case of co-infestation by these two parasites.

## Case report

**Fig. 1:**
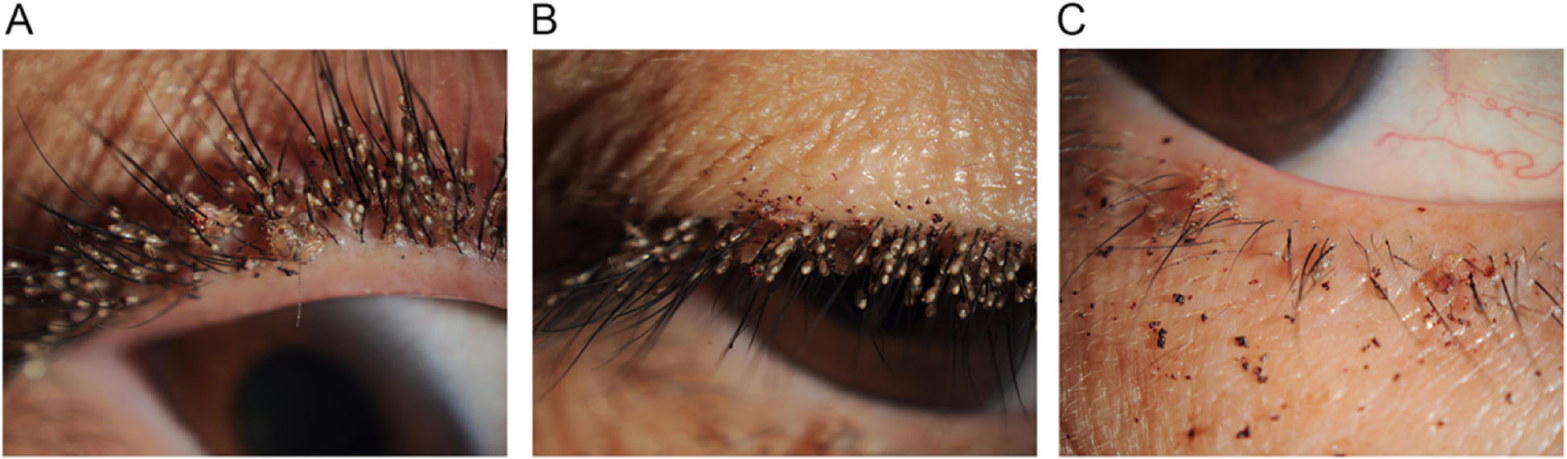
Photos of her right eye shows multiple nits and lice on eyelashes of upper lid (**a**) (**b**) and lower lid (**c**), with dried blood on skin. Under slit-lamp examination, adult lice adhere to eyelashes with crab-like bodies; the nits appear oval and translucent

A 48-year-old-woman presented to our hospital with unbearable itching and stinging with black secretion on her right eye for 2 weeks. She had no specific ocular, chronic or immune disease. Her best corrected visual acuity was 20/20. Slit lamp examination revealed over three hundred of translucent ovoid nits and over twenty live adult *P. pubis* firmly attached to the base and shaft of eyelashes. Dry blood and faeces appeared as granular and dark dots on nearby eyelids, which the patient considered as “black secretion” (Fig. 1). Lid margin vascular engorgement and mild hyperemia were observed in the conjunctiva. There was mild punctate defect on corneal epithelium. No nit, lice or any abnormal of ocular surface were observed in the left eye. The left cornea was clear and no vision decline. The patient was referred to dermatologist to detect potential phthiriasis lesions in other body parts. On dermatologic examination, lice were also detected in the pubic area. She had past history of sexual contacts with her husband. The search of sexually transmitted diseases was negative. Initial diagnosis of unilateral phthiriasis palpebrarum was made.

Mechanical removal of the lice and nits was attempted but failed as they were numerous. All eyelashes of the affected eye were trimmed with a scissors. Conjunctival sac was irrigated and lids margin was disinfected by compound iodine disinfectant cotton swab immediately and followed at weekly intervals. The patient was prescribed daily moxifloxacin eye ointment and fluorometholone eye drops four times a day for 2 weeks. The pubic hair was shaved and hexachlorocyclohexane cream was applied daily. The patient was advised to avoid close body contact and not to share clothing and towels.

**Fig. 2:**
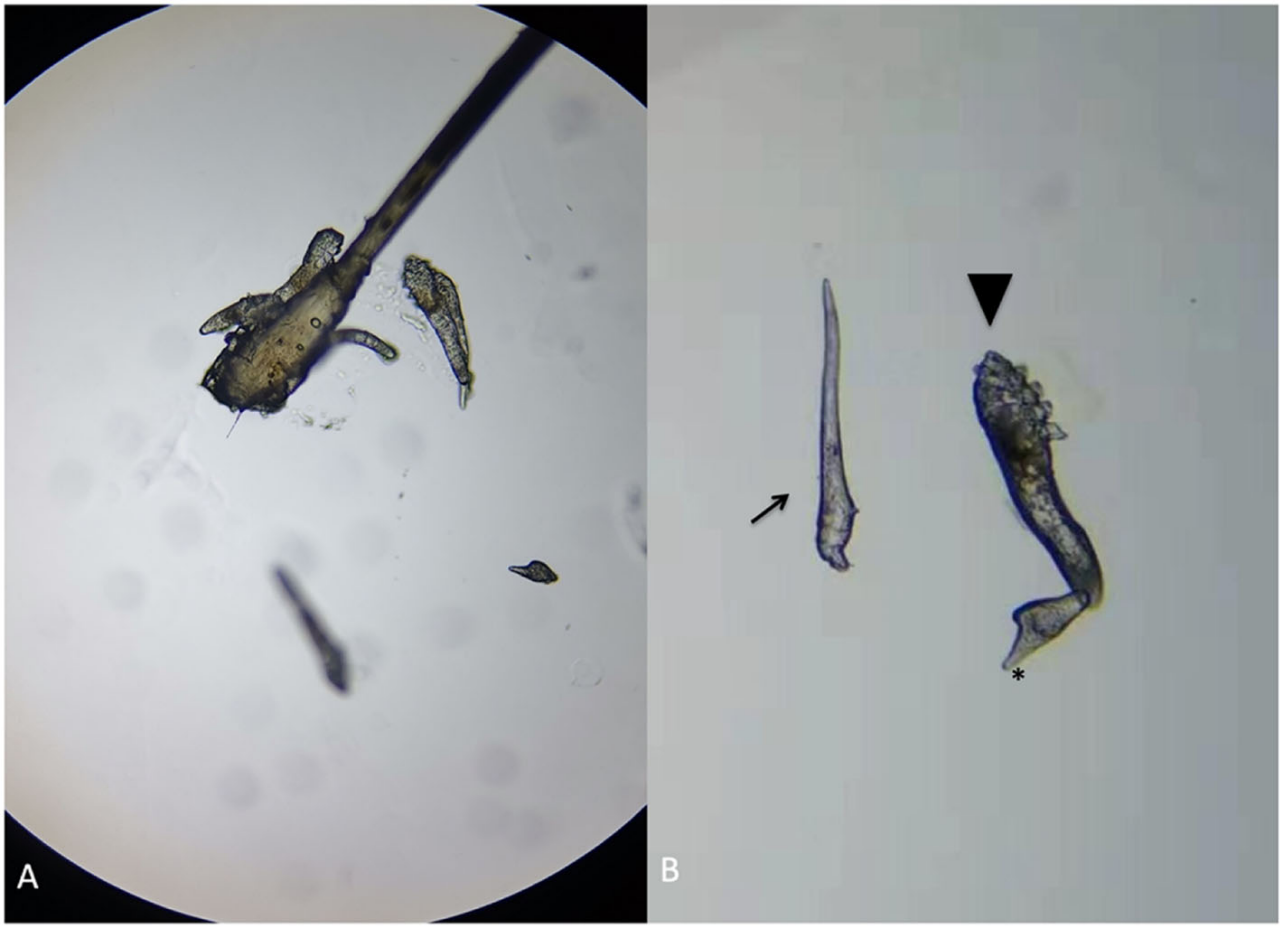
Microscopic features of Demodex in the patient’s right eye. **a** A group of Demodex and an egg were revealed with a lash follicle. **b** An egg (arrow), a larvae which had three pairs of poorly developed legs and a slender body (arrowhead), and an adult D. folliculorum with four pairs of well-developed legs (*) and a stumpier body

After two-week’s treatment, the patient returned to our hospital complaining with persistent itching of the right eye. The corneal epithelial punctate defect remained unchanged. There were no lice or nits observed on right eye. A co-infestation by *Demodex* was suspected. Three eyelashes from each eyelid of both eyes were epilated and examined with light microscopy. *Demodex* detection and counting in epilated lashes were performed as proposed by Gao et al. [3]. A total of 14 *D. folliculorum* was found in 6 lashes from the right eye (Fig. 2) and a total of 5 *D. folliculorum* was found in 6 lashes from the left eye. The diagnosis of demodicosis was confirmed. The lid margin around the root of the eyelashes were scrubbed by a sterile cotton-tipped applicator saturated with 25% tea tree oil daily and eyelids were heated with wet towel at about 40–45 °C twice per day for 2 months. Clothing, pillow cases, and towels were recommended to be washed with hot water and then heat dried for up to 10 mins. The patient’s symptoms relived, and the affected corneal was clear after two-week’s treatment. The number of mites during an examination 1 month later reduced to 2 in the right eye and 0 in the left eye, and was reduced to 0 in both eye 2 months later. No recurrence was observed during 3 months of follow-up.

## Discussion and conclusions

This is the first report of co-infestation by two different kinds of parasites in human eyelids, and also the most severe case of phthiriasis palpebrarum that has been reported in literature.

The infestation of phthiriasis palpebrarum happens mainly in people who live in crowded places or poor hygiene conditions. The infestation is usually transmitted by sexual activity because *P. pubis* is less mobile and cannot fly or jump from the initial located area to the eyes. Therefore, sexual abuse should be considered in children with *P. pubis* infestation [4]. There are multiple treatment options available like mechanical removal with forceps, pilocarpine hydrochloride, liquid Vaseline, moxifloxacin eye ointment, 1% mercury oxide, cryotherapy, argon laser, topical botulinum toxin application, 50% tea tree oil, and 20% fluorescein eye drops [5–7]. In this case, we trimmed the eyelashes and treated patient with moxifloxacin eye ointment and fluorometholone eye drops. No lice or eggs were observed on the eyelashes.

Eye infestation by *Demodex* is much more common than *P. pubis*. Among a wide range of species, only *D. folliculorum* and *D. brevis* can parasitize the human eye. *Demodex folliculorum* lives in the lash follicle, whereas *Demodex brevis* lives solitarily and deeply in the sebaceous gland of the eyelash and the meibomian gland [2]. Hence, when eyelashes are sampled, the detection rate of *D. folliculorum* is much higher than that of *D. brevis*. Patients has cylindrical dandruff in eyelash roots should highly considered *Demodex* infestation [3, 8, 9]. However, in the presenting case, there was no typical cylindrical dandruff observed in both eyes. The previous treatment of topical eye ointment and weekly lid cleaning might covered the tracks of *Demodex* and its secretion on right eye. And the number of *Demodex* infestation on the left eye is too low to cause any symptoms.

Because *Demodex* can be found in healthy asymptomatic population, some authors have suggested that the relationship between symptoms and the number of *Demodex* should be considered at the same time. Three mites per three lashes should consider as *Demodex* infestation positive. If the related symptoms of *Demodex* infestation are serious, it can be diagnosed once the mite is detected [3, 8–10]. Many methods, including tea tree oil, 1% yellow mercury ointment, 2% topical metronidazole gel, 1% acaricide permethrin, and daily lid scrubbing and cleaning, can be used for eradicating ocular *Demodex* infestation [11–13]. We used 25% tea tree oil daily and heated the eyelids with wet towel at about 40–45 °C twice per day for 2 months. The patient’s symptoms relived, and the affected corneal was clear after two-week’s treatment. No recurrence was observed during 3 months of follow-up.

Other authors have suggested that demodicosis may be associated with leukemia or immunodeficient patients with HIV-infection [14–16] or patients with end stage chronic renal failure [17]. However, the causal relationship between *P. pubis* infestation and *Demodex* infestation has not yet been studied.

In conclusion, here we report the first case of a severe infestation of *P. pubis* co-infested with *Demodex* in human eye. This report shows the importance of an early and comprehensive diagnosis, because both phthiriasis palpebrarum and demodicosis can be confused with blepharitis. In this case, the *Demodex* infestation of the right eye is much more severe than the left eye, and *P. pubis* only infested in the right eye. Whether the host’s immune reactions by *Demodex* make it susceptible for *P. pubis* infestation is still unclear.

## Data Availability

All data generated or analyzed during this study are included in this published article.
